# Supplementary material for: A novel assay to measure low-density lipoproteins binding to proteoglycans
Source: PLoS One. 2024 Jan 31;19(1):e0291632. doi: 10.1371/journal.pone.0291632 (PMC10830033; doi:10.1371/journal.pone.0291632)
Supplement: S1 File — (PDF) [file pone.0291632.s001.pdf]

A novel assay to measure low-density lipoproteins binding to proteoglycans

Esmond N Geh[1*], Debi K Swertfeger[1], Hannah Sexmith[1], Anna Heink[1], Pheruza Tarapore[2], John T Melchior[2,3,4], W. Sean W Davidson[2] and Amy Sanghavi Shah[1].

[1]Division of Endocrinology, Cincinnati Children's Hospital Medical Center & the Department of Pediatrics, University of Cincinnati College of Medicine, Cincinnati, OH

[2]Center for Lipid and Arteriosclerosis Science, Department of Pathology and Laboratory Medicine, University of Cincinnati, Cincinnati, OH 45237-0507, USA.

[3]Biological Sciences Division, Pacific Northwest National Laboratory, Richland, Washington 99354, USA.

[4]Department of Neurology, Oregon Health and Science University, Portland, Oregon 97239, USA.

*Corresponding author

Email: Esmond.geh@cchmc.org

SUPPLEMENTAL INFORMATION

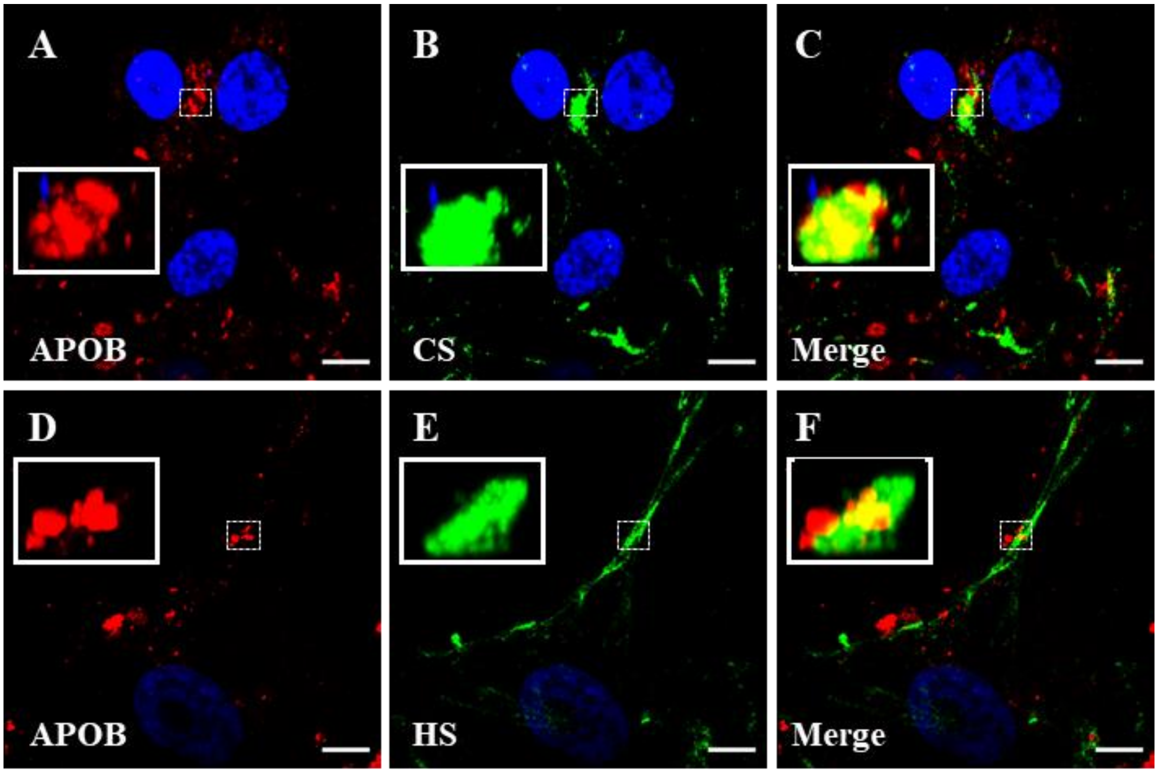

S1 Fig

SI Fig. LDL Colocalizes with CS and HS proteoglycans in Human ascending aorta smooth muscle (HAAVAS) cells. Maximum intensity projection (MIP) of a z-stack images of MOVAS cells labeled with APOB (**A, D**), *red*; CS (**B**), and HS (**E**), *green*. DAPI, used to stain nuclei, is shown in *blue*. **C** and **F** represent merged MIP images for CS and HS, while *inserts* in the upper right display an enlarged 3D rendition of regions of interest (*dotted box*) within the respective images. Colocalized regions are shown in yellow. Scale bars indicate 10 µM. *Abbreviations; CS-chondroitin sulfate, HS-heparan sulfate*.

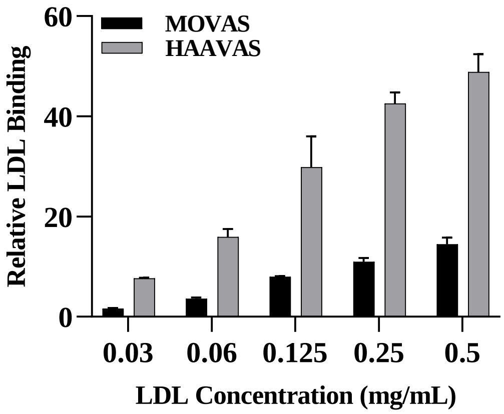

S2 Fig

S2 Fig. LDL binds ECM of MOVAS and HAAVAS cells in a dose-dependent manner. Mouse aorta vascular smooth muscle (MOVAS) cells (*black bars*) and human ascending aorta smooth muscle (HAAVAS) cells (*grey bars*) were preincubated with various concentrations of LDL. ICE was performed and the bound LDL signal was calculated by subtracting the background signal (empty wells with corresponding amount of LDL) from the experimental signals (wells with cells). Results are expressed as relative LDL binding in arbitrary units (light units divided by cellular DNA content measured by Janus Green B). The mean and standard deviation (n=4) are shown. Unpaired T-tests show a statistical significant difference of $p<0.001$ between HAAVAS and MOVAS cells.

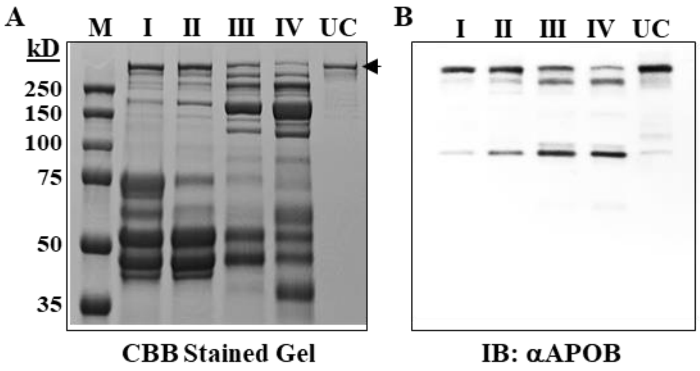

S3 Fig

S3 Fig. ICE assay detects differences in PG binding of various LDLs. CBB stained gel **(A)** and Western blot using αAPOB antibody **(B)** of FPLC purified LDL subfractions. Black arrows indicate the expected location of APOB band. *Abbreviations: CBB-Coomasie brilliant blue; Kd-kilodalton; M-Marker; I-IV-FPLC purified LDL fractions; UC-ultracentrifugally isolated LDL, IB-Immunoblot.*

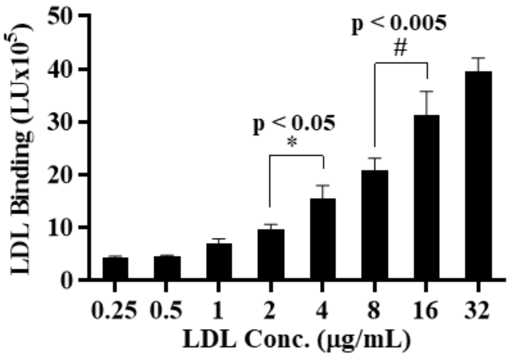

S4 Fig

S4 Fig. Increased sensitivity of LDL binding is obtained by using biotinylated APOB detected with HRP-conjugated streptavidin. MOVAS cells were preincubated with various concentrations of LDL. Results are expressed as LDL binding in light units. Bound LDL signal was calculated by subtracting the background signal (empty wells with corresponding amount of LDL) from the experimental signals (wells with cells). The mean and standard deviation (n=4) are shown. Paired T-test show a significant difference (*$p<0.05$, $^{\#}p<0.005$) between LDL treated and untreated samples.
