# Supplementary figures and images for: A novel assay to measure low-density lipoproteins binding to proteoglycans
Source: PLoS One. 2024 Jan 31;19(1):e0291632. doi: 10.1371/journal.pone.0291632 (PMC10830033; doi:10.1371/journal.pone.0291632)

## Original Figure 6A

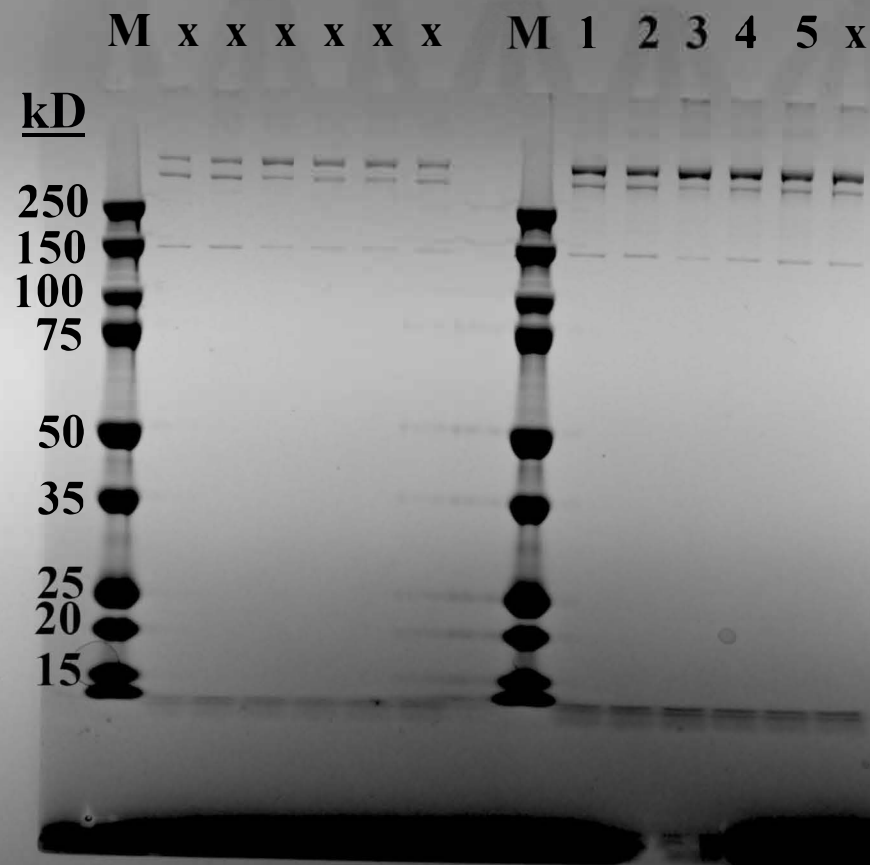

Original Figure 6C

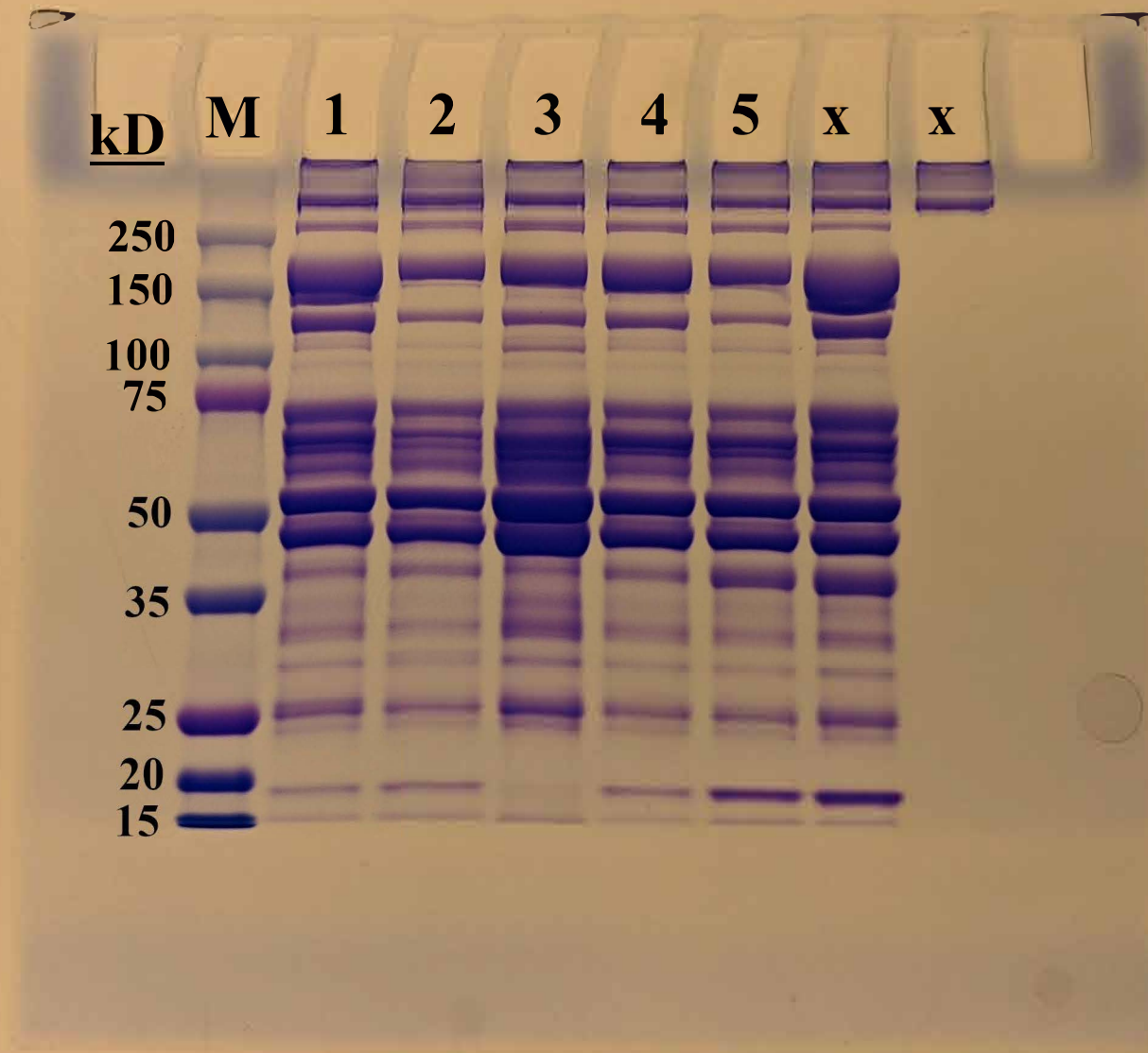

Original Figure 6E

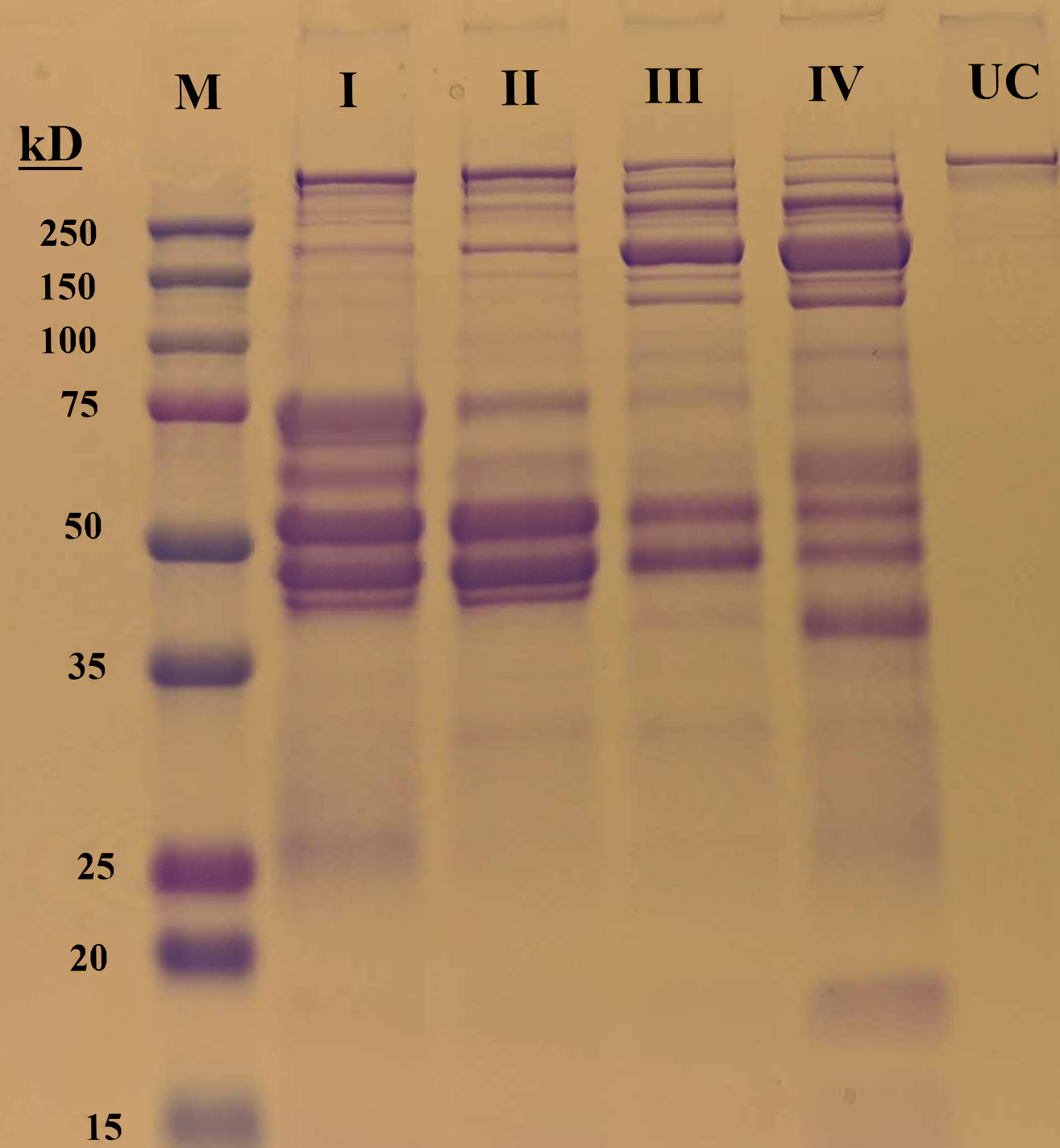

Original Figure S3A

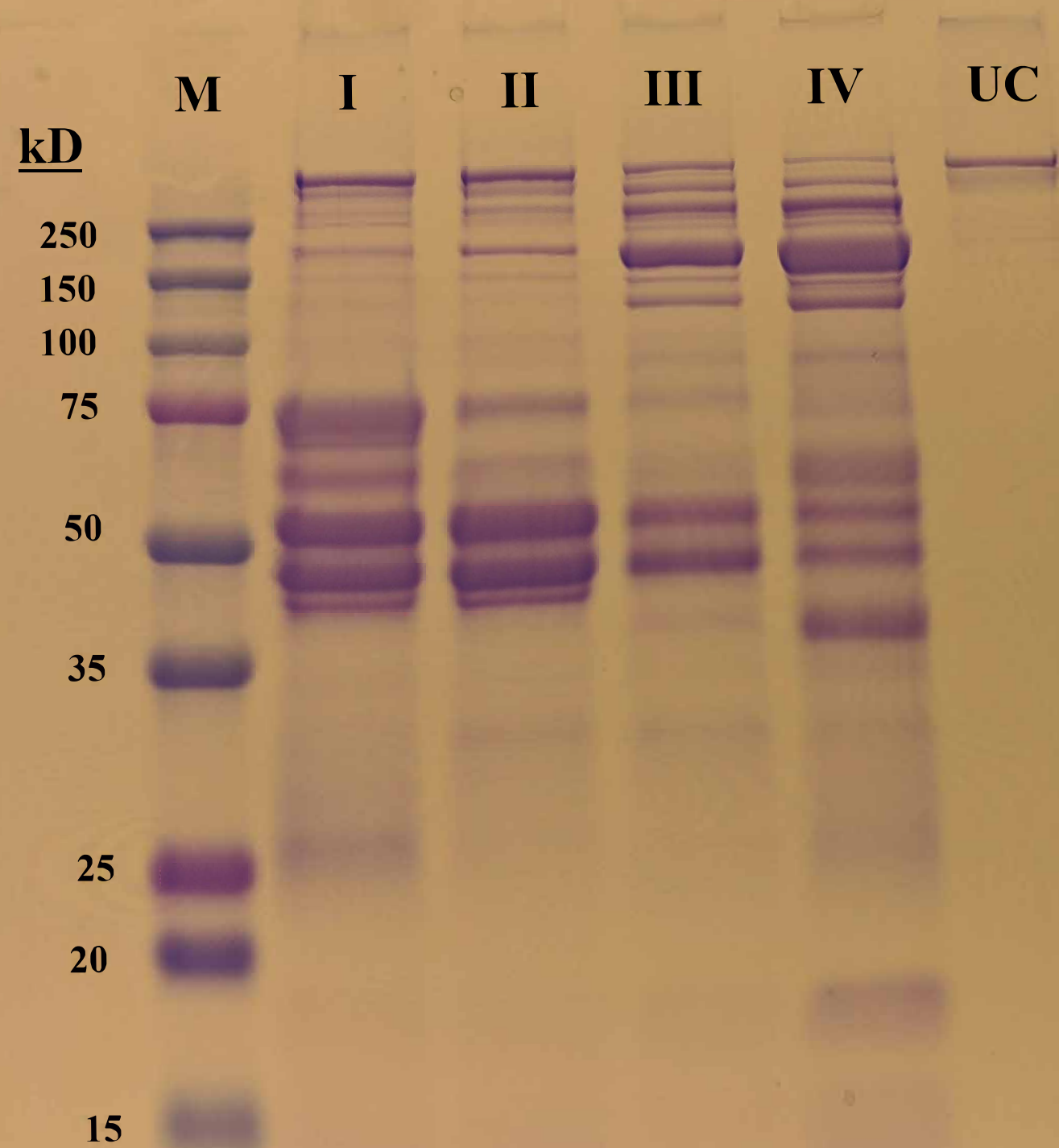

# Original Figure S3B

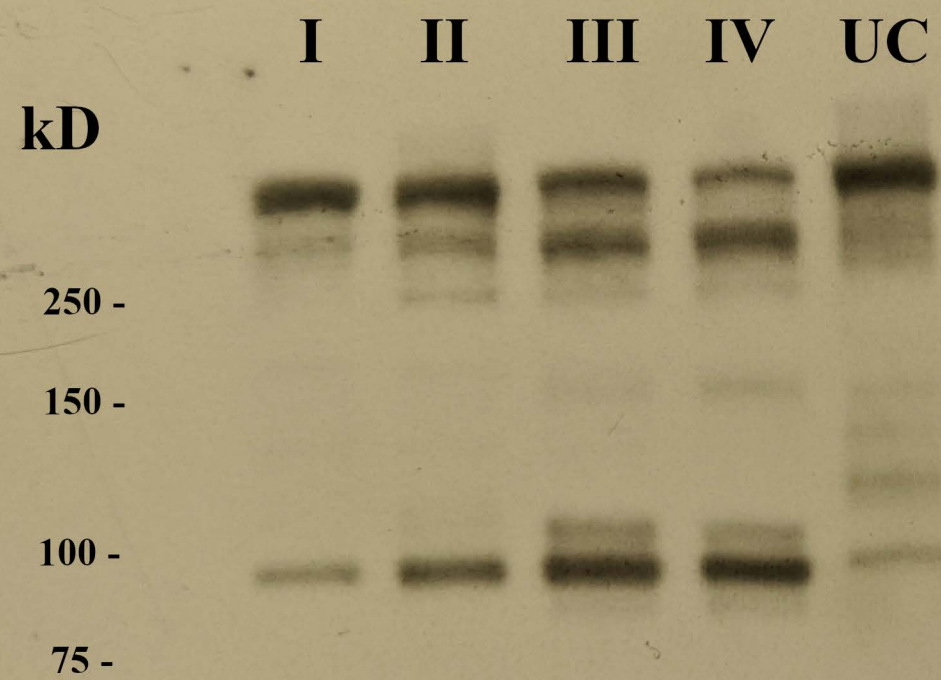

Supplement: S1 Raw images — (PDF) [file pone.0291632.s003.pdf]

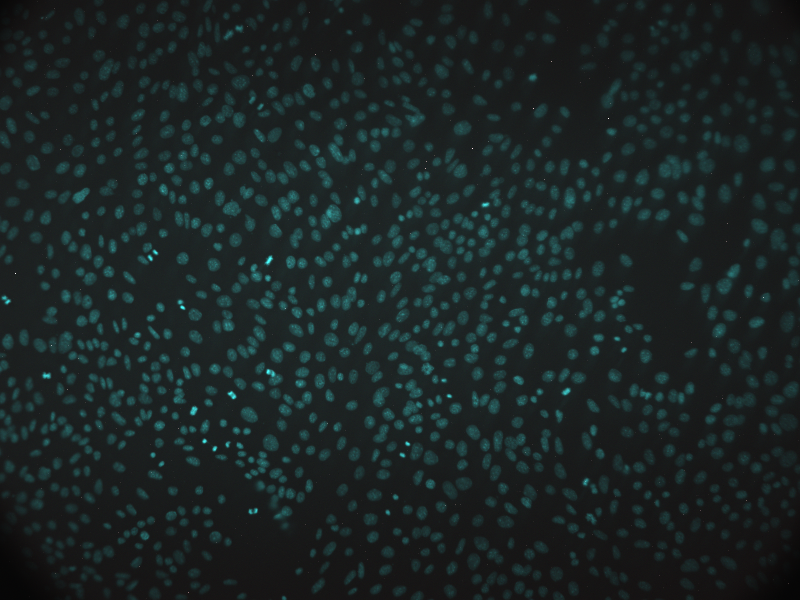

Supplement: S2 Raw images — (ZIP) [file pone.0291632.s004.zip › Fig_2/Fig 2A.TIF]

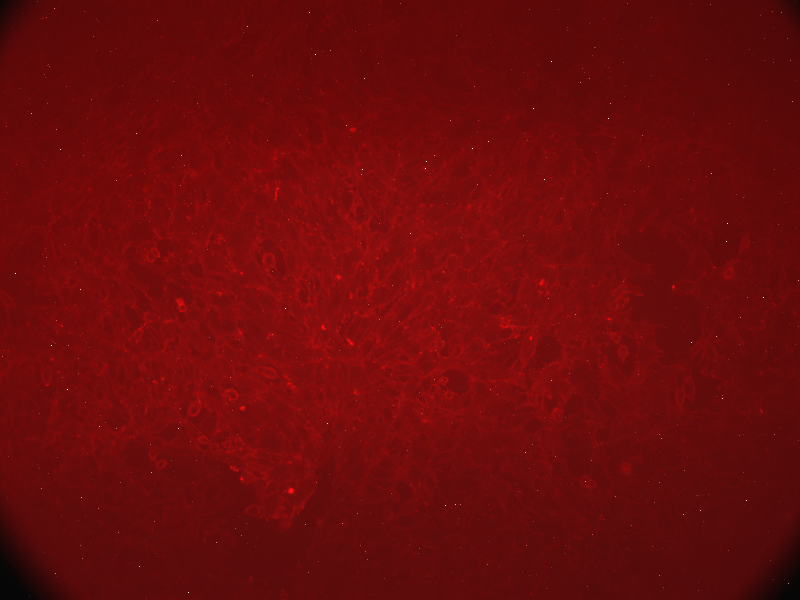

Supplement: S2 Raw images — (ZIP) [file pone.0291632.s004.zip › Fig_2/Fig 2B.TIF]

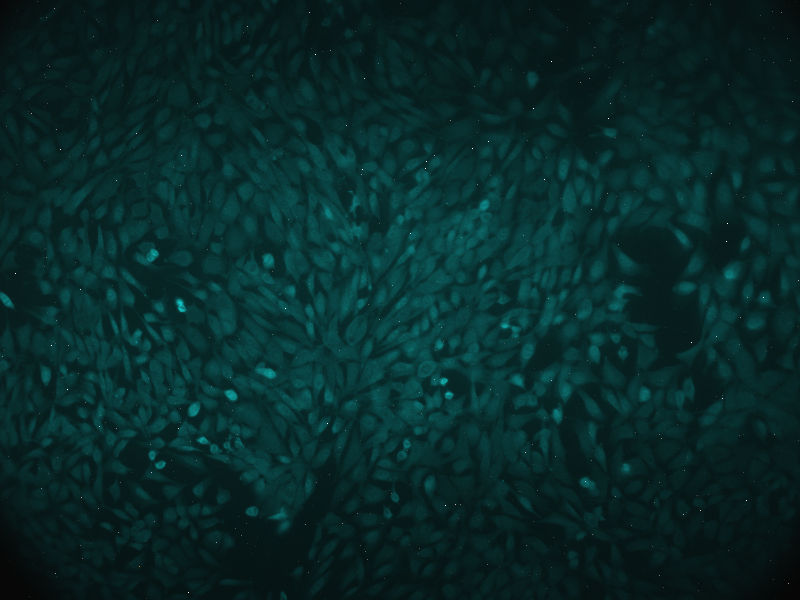

Supplement: S2 Raw images — (ZIP) [file pone.0291632.s004.zip › Fig_2/Fig 2C.TIF]

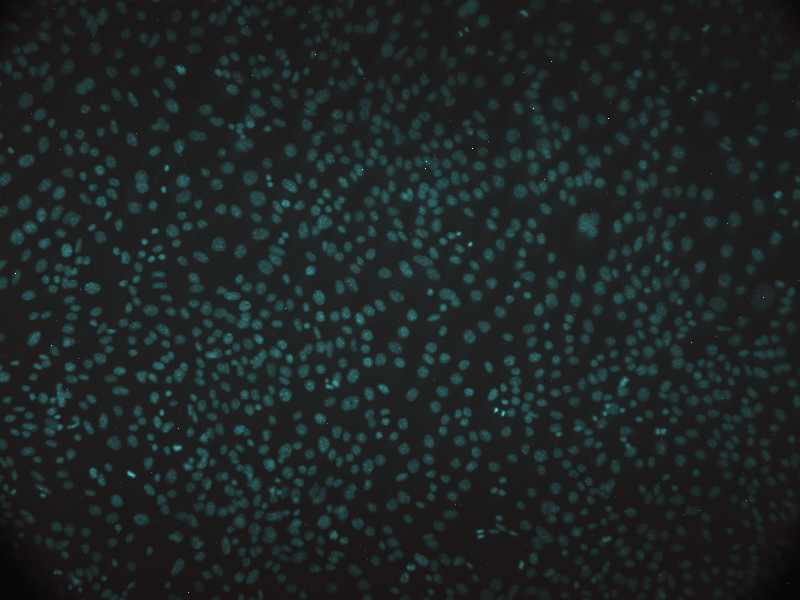

Supplement: S2 Raw images — (ZIP) [file pone.0291632.s004.zip › Fig_2/Fig 2E.TIF]

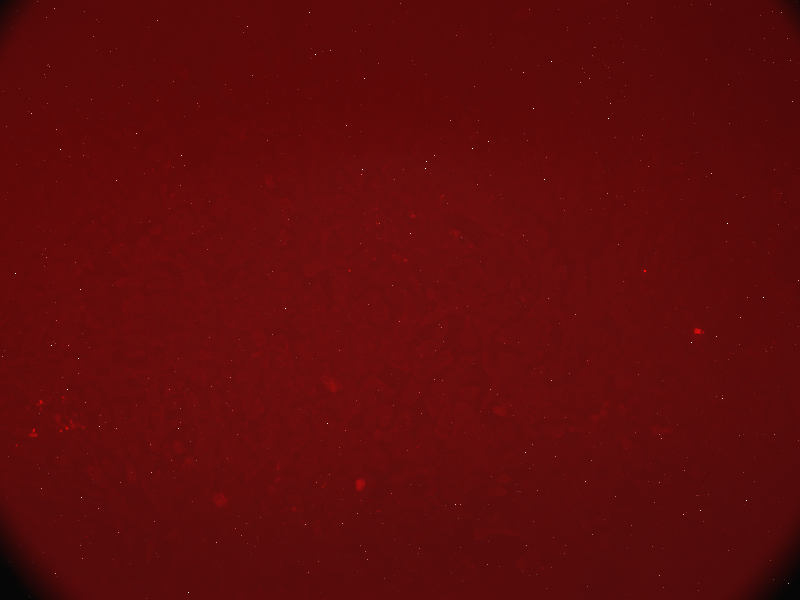

Supplement: S2 Raw images — (ZIP) [file pone.0291632.s004.zip › Fig_2/Fig 2F.TIF]

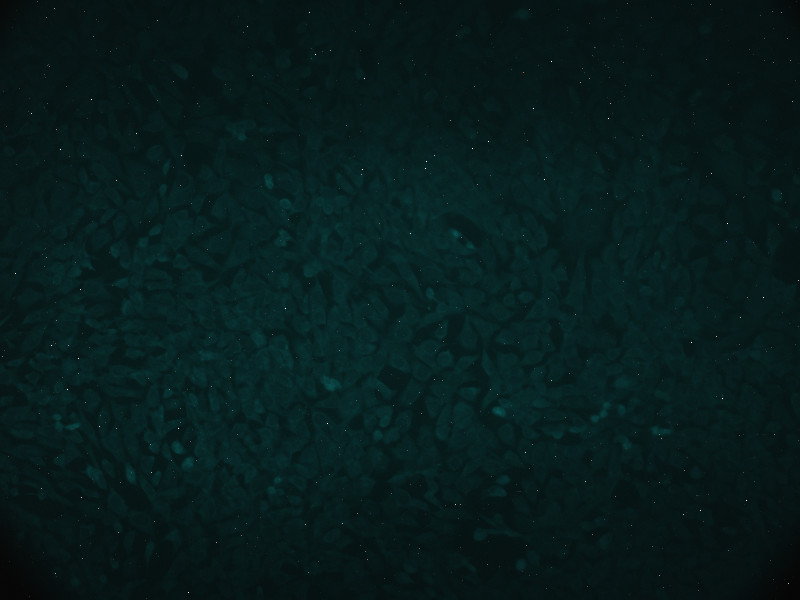

Supplement: S2 Raw images — (ZIP) [file pone.0291632.s004.zip › Fig_2/Fig 2G.TIF]
